# Discovery of circulating miRNAs as biomarkers of chronic Chagas heart disease via a small RNA-Seq approach

**DOI:** 10.1038/s41598-024-51487-9

**Published:** 2024-01-12

**Authors:** Silvina R. Villar, Alfonso Herreros-Cabello, Francisco Callejas-Hernández, María C. Maza, Javier del Moral-Salmoral, Mario Gómez-Montes, Héctor O. Rodríguez-Angulo, Irene Carrillo, Miguel Górgolas, Pau Bosch-Nicolau, Israel Molina, José A. Pérez-Molina, Begoña Monge-Maillo, Oscar A. Bottasso, Juan Beloscar, Ana R. Pérez, Manuel Fresno, Núria Gironès

**Affiliations:** 1 Instituto de Inmunología Clínica y Experimental de Rosario (IDICER-CONICET-UNR), Rosario, Argentina; 2 Departamento de Biología Molecular, Universidad Autónoma de Madrid (UAM), 28049 Madrid, Spain; 3 Centro de Biología Molecular Severo Ochoa (CSIC-UAM), Madrid, Spain; 4 Instituto Venezolano de Investigaciones Científicas- IVIC, Caracas, 1021 Venezuela; 5 Division of Infectious Diseases, IIS-Fundación Jiménez Díaz, Madrid, Spain; 6 Department of Medicine, Universidad Autónoma de Madrid, Madrid, Spain; 7 International Health Unit Vall d’Hebron-Drassanes, Infectious Diseases Department, Vall d’Hebron University Hospital, PROSICS Barcelona, Barcelona, Spain; 8 Centro de Investigación Biomédica en Red de Enfermedades Infecciosas (CIBERINFEC), Instituto de Salud Carlos III, Madrid, Spain; 9 National Referral Unit for Tropical Diseases, Infectious Diseases Department, Ramón y Cajal University Hospital, IRICYS, Madrid, Spain; 10 CIBER de Enfermedades Infecciosas, Instituto de Salud Carlos III, Madrid, Spain; 11 Cátedra y Servicio de Cardiología, Sección Chagas, Hospital Provincial del Centenario, Rosario, Argentina; 12 Instituto Universitario de Biología Molecular, Universidad Autónoma de Madrid (IUBM-UAM), Madrid, Spain; 13 Instituto de Investigación Sanitaria, Hospital Universitario de La Princesa, Madrid, Spain; 14 Present Address: Center for Genomics and Systems Biology, Department of Biology, New York University, New York, NY USA

**Keywords:** Infectious-disease diagnostics, Parasitology, Pathogens, Microbiology, Molecular biology, Biomarkers, Cardiology, Diagnosis, Infection

## Abstract

Chagas disease affects approximately 7 million people worldwide in Latin America and is a neglected tropical disease. Twenty to thirty percent of chronically infected patients develop chronic Chagas cardiomyopathy decades after acute infection. Identifying biomarkers of Chagas disease progression is necessary to develop better therapeutic and preventive strategies. Circulating microRNAs are increasingly reliable biomarkers of disease and therapeutic targets. To identify new circulating microRNAs for Chagas disease, we performed exploratory small RNA sequencing from the plasma of patients and performed de novo miRNA prediction, identifying potential new microRNAs. The levels of the new microRNAs temporarily named miR-Contig-1519 and miR-Contig-3244 and microRNAs that are biomarkers for nonchagasic cardiomyopathies, such as miR-148a-3p and miR-224-5p, were validated by quantitative reverse transcription. We found a specific circulating microRNA signature defined by low miR-Contig-3244, miR-Contig-1519, and miR-148a-3 levels but high miR-224-5p levels for patients with chronic Chagas disease. Finally, we predicted in silico that these altered circulating microRNAs could affect the expression of target genes involved in different cellular pathways and biological processes, which we will explore in the future.

## Introduction

Chagas disease is caused by a flagellate parasite, *Trypanosoma cruzi (T. cruzi)*, first described in 1909 in a patient by Carlos Chagas. The World Health Organization estimates that approximately 7 million people are infected worldwide, a serious health problem in Latin America and nonendemic countries in North America (Mexico, Canada, and the United States), Europe (Spain), and the Western Pacific Region (Australia and Japan)^1^.

The course of Chagas disease comprises two phases, the acute phase that lasts 2–4 months, followed by a chronic phase that can be categorized into indeterminate, cardiac, and/or digestive stages with different clinical manifestations^2^. Only 20–30% of patients develop symptoms after years to decades of clinical latency (indeterminate stage), showing no detectable parasitemia in microscopic evaluations at this stage. Myocardial damage in Chagas disease is a progressive process that can be classified according to the degree of myocardial involvement^3^.

MicroRNAs (miRNAs) are a class of small noncoding RNAs (20–25 nucleotides) involved in gene regulation and expression at the posttranscriptional level produced by cells that can be found in extracellular vesicles and blood^4,5^. miRNA transcription depends on RNA polimerase II, which generates a primary transcript (pri-miRNA) with hairpin structure. This pri-miRNA is processed by the Drosha complex to form the pre-miRNA that will be transported to the cytoplasm through the exportin-5. Then, it will be recognized and cut by Dicer and RNA-induced silencing complex (RISC) proteins to create the mature miRNA^6^. miRNAs are key for a set of molecular signaling pathways and cellular pathophysiological effects of different but interrelated disorders, such as cardiovascular diseases, cardiac hypertrophy, coronary heart disease, myocardial infarction^7–11^, infections, type 1 diabetes, inflammatory, infectious and autoimmune processes, and cardiovascular risk factors^12–15^. An advantage is that changes in miRNA levels in body fluids occur earlier than conventional biomarkers^16^. This makes them promising potential candidates as prognostic biomarkers of disease.

miRNAs may play a major role in the control of gene expression in key pathological processes in chronic Chagas cardiomyopathy (CC)^5^. However, more studies are needed to understand the role of miRNAs in CC and there are only a few studies on miRNAs as biomarkers in this disease. These studies used quantitative reverse transcription PCR (RT‒qPCR) for specific miRNAs and suggested a role in the pathogenesis, diagnosis, and prognosis of Chagas disease^17,18^. Circulating miR-208a, a heart-specific miRNA playing a critical role in heart failure, was increased during the chronic indeterminate phase when compared to chronic cardiac clinical forms in Chagas disease individuals^17^. Additionally, circulating miR-19a-3p, miR-21-5p, and miR-29b-3p, previously associated with heart failure, cardiac fibrosis, and hypertrophy, were increased in chronic cardiac Chagas disease and correlated with cardiac injury^18^. In addition, other authors described the dysregulation of different miRNAs in the cardiac tissue of patients with chronic Chagas cardiomyopathy^19^.

Considering the urgent need of biomarkers in CC, and the absence of studies addressing the role of miRNAs in CC, in the present study, we aimed for the first time to identify new circulating miRNAs using an exploratory small RNA sequencing (Small RNA-Seq) approach and posterior RT-qPCRs in the plasma of chagasic patients at various stages of the disease. Therefore, these miRNAs could be used as biomarkers of chronic Chagas cardiomyopathy and likely involved in its development.

## Results

### Small RNA-Seq alignment and analysis

To explore the circulating miRNA transcriptomic profile in chagasic patients, we performed exploratory small RNA-Seq of pools of miRNAs extracted from the plasma of healthy controls (HC), indeterminate Chagas disease (ICD), and mild and moderate Chagas cardiomyopathy (MCC) patients (n = 5 each) attending to the *Hospital Provincial del Centernario* (HPC, Rosario, Argentina). We aligned the reads to the human genome (*H. sapiens*, GRch38, version 25/03/2019) using Bowtie 2. The mean percent alignment of total reads aligned at least once to the human genome was 98.95% (Table S2). Then, reads aligning to known miRNAs were determined using miRBase annotations (http://www.mirbase.org/ftp.shtml) and normalized (Supp. Data S2).

An initial analysis was performed to check whether we could find altered levels of miRNAs in the chagasic patients compared to the HCs. We performed a preliminary PCA study with normalized reads (Fig. S1). PC components explained 100% of the variance of the data. As expected, ICD and MCC grouped separately from HC individuals, indicating that we would probably find candidate miRNAs for biomarkers.

### Identification of new miRNAs

To identify new miRNAs in the small RNA-Seq, we used miRDeep2. We identified several potential new miRNAs (Table S3, column 2). miRDeep2 considers the loop sequence and structure compatible with Dicer processing as well as the conservation of the seed sequence (among other stringent parameters) to predict sequences of potentially new miRNAs and structures.

Potential new miRNAs identified in chagasic patients with at least 80% similarity were grouped into clusters, which are defined as potential precursors of miRNAs and/or mature miRNAs separated in the genome from other clusters by a minimum of 30 nucleotides^20^ (Table S3, column 3).

**Figure 1:**
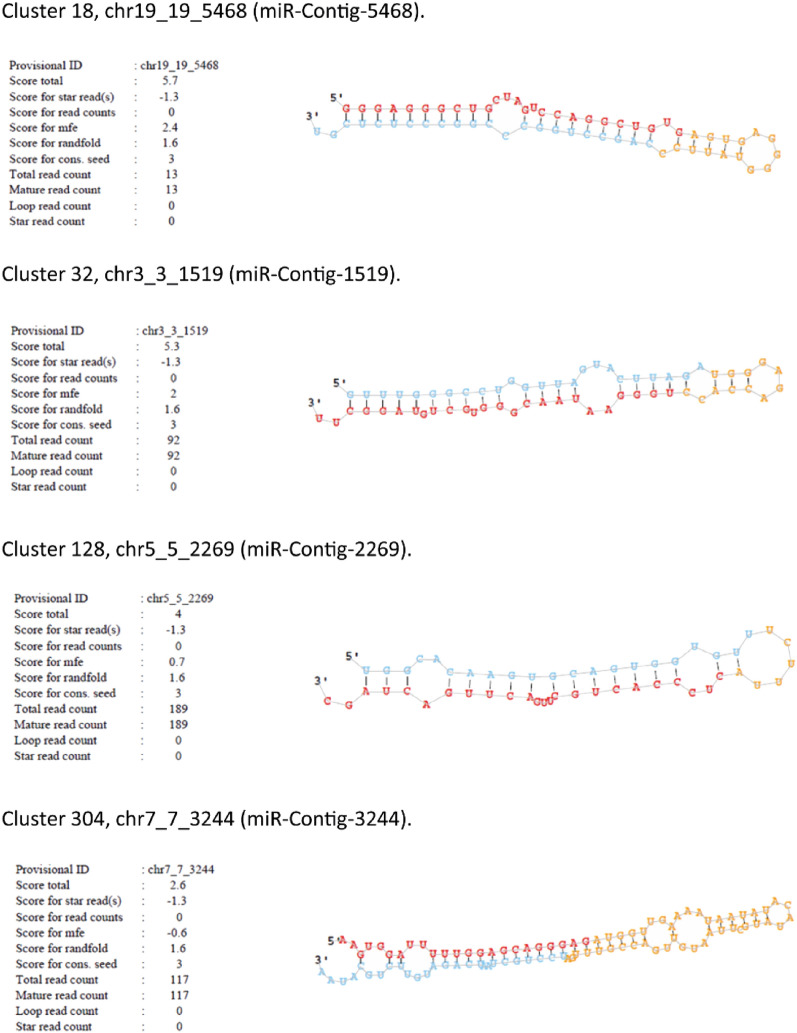
Predicted secondary structure of the hairpins corresponding to the new miRNAs using miRDeep2. Red: mature miRNA, blue: star sequence, orange: loop.

Then, data were filtered according to star sequence, precursor, and loop structures, and only those with significant ranfold p-values were considered (Table S4). These short sequences showed a total score and secondary structure consistent with valid mature miRNA sequences, suggesting that they could be considered potential new miRNAs. Moreover, using miRDeep2, we predicted the secondary structure of these 4 new miRNAs and validated them with the precursor structure (Fig. 1).

Most canonical miRNAs are generated from long noncoding regions, and a miR gene can generate more than one mature miRNA able to target different genes (isomiRs). However, although less frequently, miRNAs can also be generated from coding regions^21^. Three of the new miRNAs mapped to the following regions of the genome: miR-Contig-5468 to *Homo sapiens* CACTIN antisense RNA 1 (CACTIN-AS1), long noncoding RNA (from RefSeq NR_038865); miR-Contig-2269 to *Homo sapiens* SREK1 interacting protein 1 (SREK1IP1), mRNA (from RefSeq NM_173829); and miR-Contig-3244 to RNA, U2 small nuclear 29, pseudogene (from HGNC RNU2-29P), while miR-Contig-1519 mapped to a noncoding region.

### Validation by RT‒qPCR of the new circulating miRNAs

**Figure 2:**
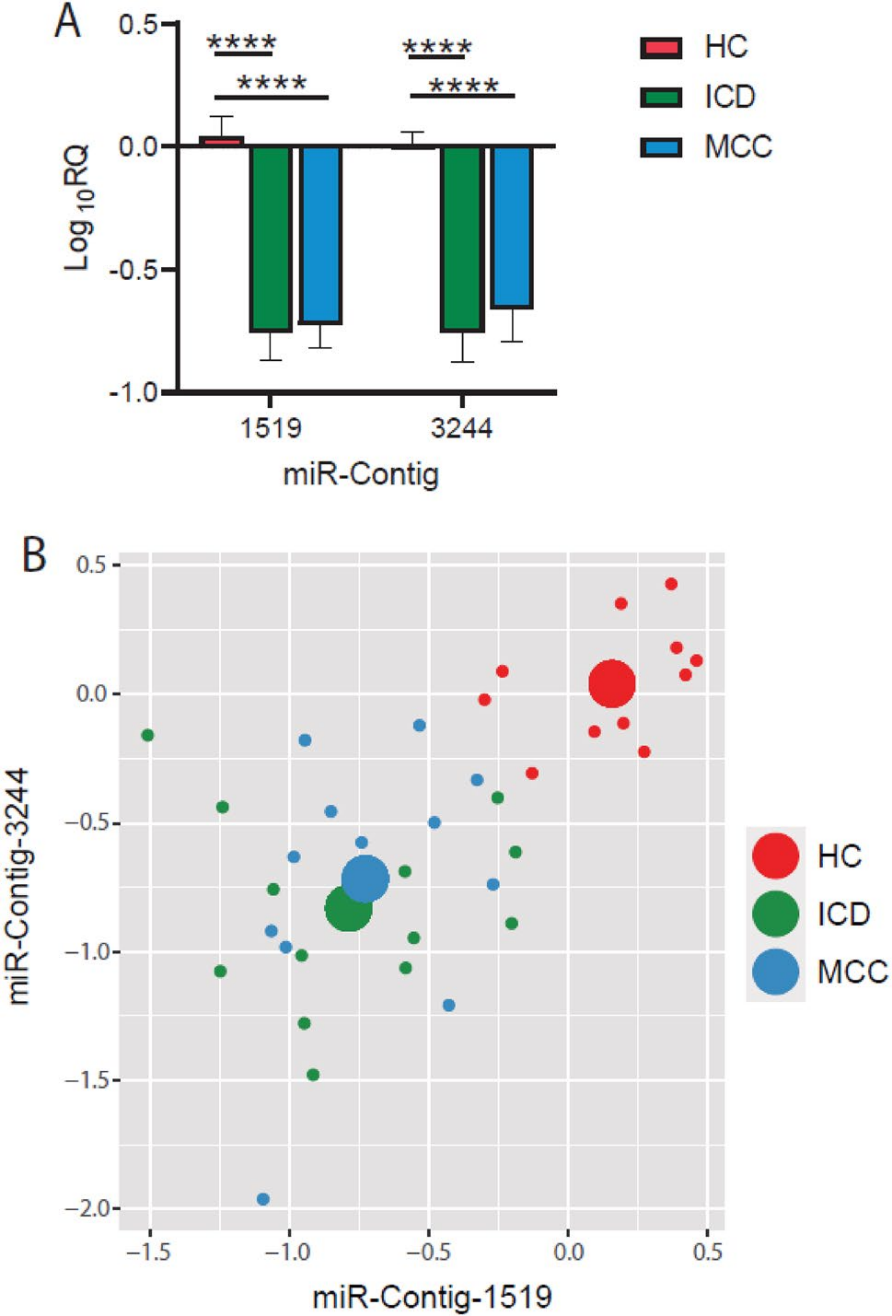
RT-qPCR analysis of new circulating miRNAs. (**A**) Quantification of the levels of miRNA in serum/plasma samples from FJD and HVH patients. After the Shapiro‒Wilk normality test, two-way ANOVA tests were applied (*p<0.05, **p<0.01, ***p<0.001, ****p<0.0001). (**B**) Plot of HC, ICD, and MCC individuals considering miR-Contig-1519 and miR-Contig-3244 data.

To validate the presence of these newly predicted miRNAs in humans, we studied their expression in serum/plasma samples from patients from *Fundación Jiménez Díaz* (FJD, Madrid, Spain) and Vall d'Hebrón University Hospital (HVH, Barcelona, Spain). Only 2 out of 4 were confirmed: miR-Contig-1519 and miR-Contig-3244 were detected, while miR-Contig-2269 and miR-Contig-5468 were not detected by RT‒qPCR (Fig. 2A). Both miR-Contig-1519 and miR-Contig-3244 showed a marked decrease in chagasic asymptomatic and symptomatic patients of all the hospitals compared to healthy controls. Considering this result, we performed an analysis to study whether these new miRNAs could serve to discriminate patients with chronic Chagas disease from healthy individuals (Fig. 2B). The results showed that both ICD and MCC individuals grouped and separated from the HC subjects. Thus, all these results point to miR-Contig-1519 and miR-Contig-3244 as possible biomarkers of chronic Chagas disease, reducing their expression in patients.

### Levels of known circulating miRNAs of interest by RT‒qPCR

After the analysis of new miRNAs, we also decided to perform a study of known miRNAs, with more than 100 normalized reads, searching for previous relationships with cardiovascular diseases in databases to complete our results. We decided to focus on some miRNAs that had been described as possible biomarkers of cardiomyopathy in other disease contexts but not in Chagas disease: miR-148a-3p, miR-181a-5p, and miR-224-5p.

**Figure 3:**
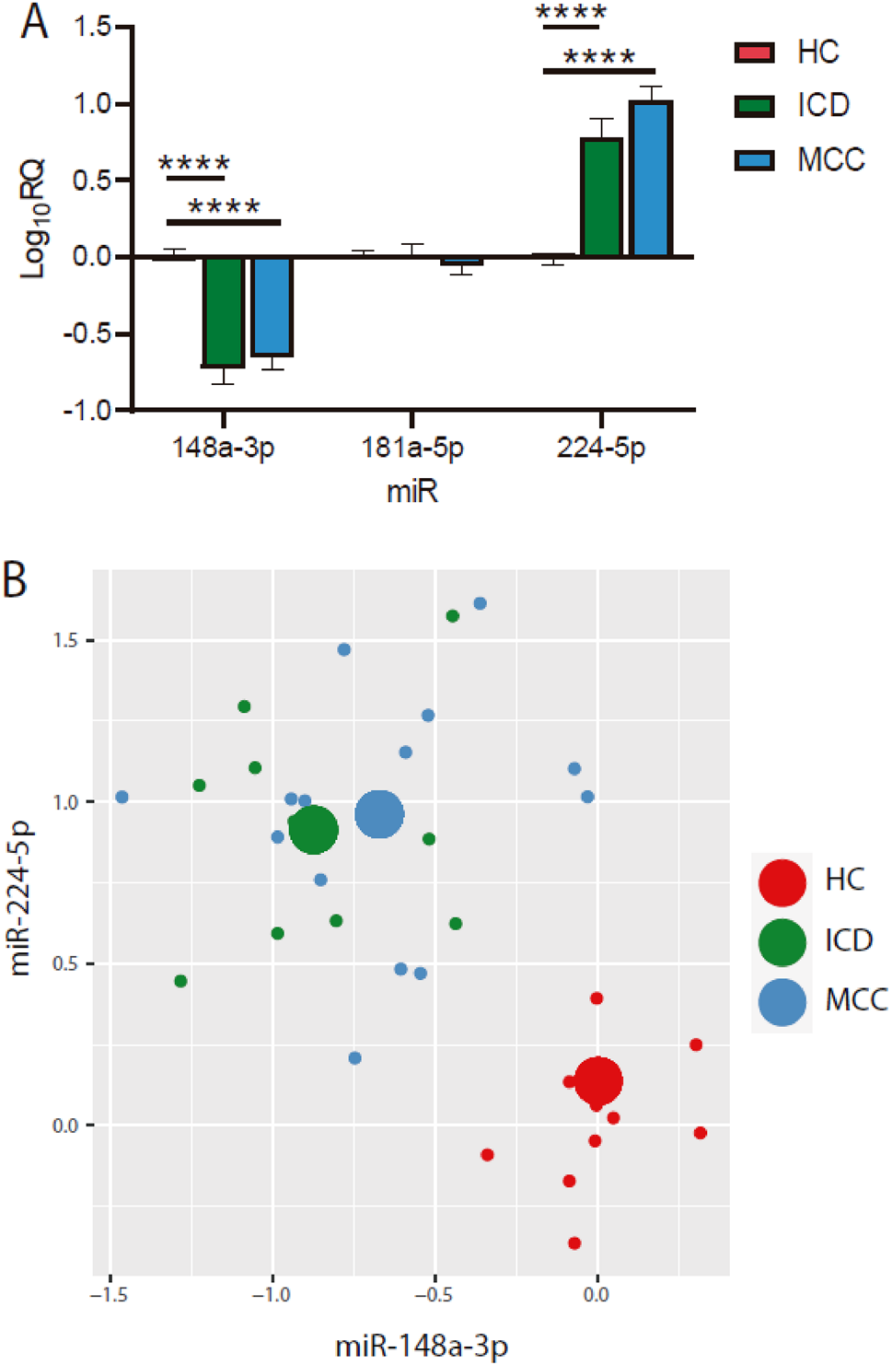
RT‒qPCR analysis of known circulating miRNAs. (**A**) Quantification of the levels of miRNAs in plasma/serum samples from FJD and HVH patients. After the Shapiro‒Wilk normality test, two-way ANOVA tests were applied (*p<0.05, **p<0.01, ***p<0.001, ****p<0.0001). (**B**) Plot of HC, ICD, and MCC individuals considering miR-148a-3p and miR-224-5p data.

To investigate the potential of those circulating miRNAs as biomarkers of chronic Chagas disease, we performed RT‒qPCR in samples from patients of different Spanish hospitals (FJD and HVH), including HC, ICD, and MCC individuals (Fig. 3A). Interestingly, miR-148a-3p decreased significantly in both ICD and MCC subjects with respect to the HC. Otherwise, miR-244-5p showed a remarkable and significant increase in ICD and MCC. However, miR-181a-5p did not display any difference between the HC and ICD/MCC groups; hence, this miRNA has no potential as a biomarker. Furthermore, considering both the miR-148a-3p and miR-224-5p results, we performed an analysis to study whether these miRNAs could serve to discriminate patients with chronic Chagas disease from healthy individuals (Fig. 3B). The results displayed a clear difference between ICD and MCC patients grouped and separated from healthy individuals, indicating that these miRNAs could serve as discriminants for chronic Chagas disease patients.

**Figure 4:**
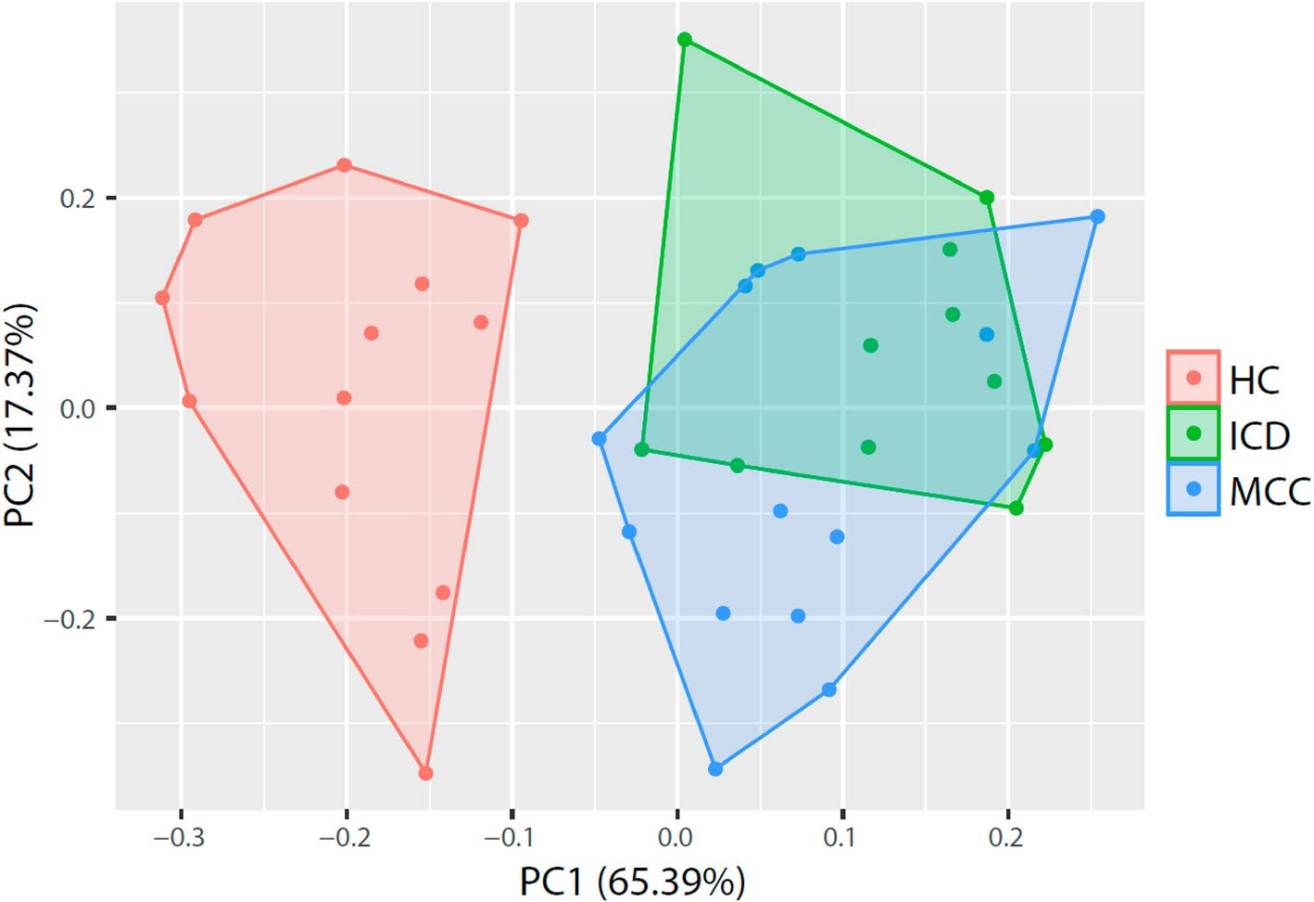
PCA of healthy controls and chronic Chagasic patients. PCA study of HC, ICD, and MCC individuals considering miR-148a-3p, miR-224-5p, miR-Contig-1519, and miR-Contig-3244 data. PC components explain 82.8% of the variance.

Considering these data and the previous resuts of the new circulating miRNAs (miR-Contig-1519 and miR-Contig-3244) that could also serve as biomarkers of Chagas disease we performed a principal component analysis (PCA) to study whether miR-148a-3p, miR-224-5p, miR-Contig-1519, and miR-Contig-3244 could be used together as a panel of biomarkers to discriminate chronic chagasic patients from healthy controls (Fig. 4). The results showed that both ICD and MCC individuals are different from HCs, although there were no differences between them, suggesting that these four miRNAs may serve to discriminate chronic Chagasic patients, independent of the presence or absence of symptoms.

### Target genes and pathways predicted for miR-Contig-1519, miR-Contig-3244, miR-148a-3p and miR-224-5p

From the above results, we defined a potential signature defined by low miR-Contig-3244, miR-Contig-1519, and miR-148a-3p and high miR-224-5p levels in chronic Chagas disease and cardiomyopathy (ICD and MCC). However, to explore the possible value of these miRNAs in terms of their contribution to the pathology of chronic Chagas disease, we used bioinformatics tools to predict the possible target genes of these miRNAs and the corresponding pathways in which they could be involved. Using miRDB, we predicted the target genes for these miRNAs (http://www.mirdb.org) (Supporting Data S3). We used the Target Search tool to determine the targets of the known miR-148a-3p and miR-224-5p that are present in the database of miRDB. For the new miRNAs, miR-Contig-1519 and miR-Contig-3244, we used the Custom Prediction tool that, according to the miRNA sequence, predicts the more likely targets. Then, we considered those targets with a score equal to or higher than 90 out of 100 and performed enrichment analysis using the EnrichR website (BioPlanet and Reactome databases).

The EnrichR web tool allows enrichment analysis according to different databases to associate gene lists with specific diseases or pathways and returns an adjusted p value of the Fisher exact test after Benjamini‒Hochberg correction^22,23^. We used the BioPlanet database since it integrates pathway annotations from available, manually curated sources with consistency and redundancy cross-evaluation^24^ and the Reactome database, which provides a broad range of physiological and pathological biological processes in humans manually curated from the primary literature and peer-reviewed manuscripts^25^. The complete list of predicted significant pathways or biological processes likely dependent on these miRNAs of the signature is shown in Supporting Data S4.

**Figure 5:**
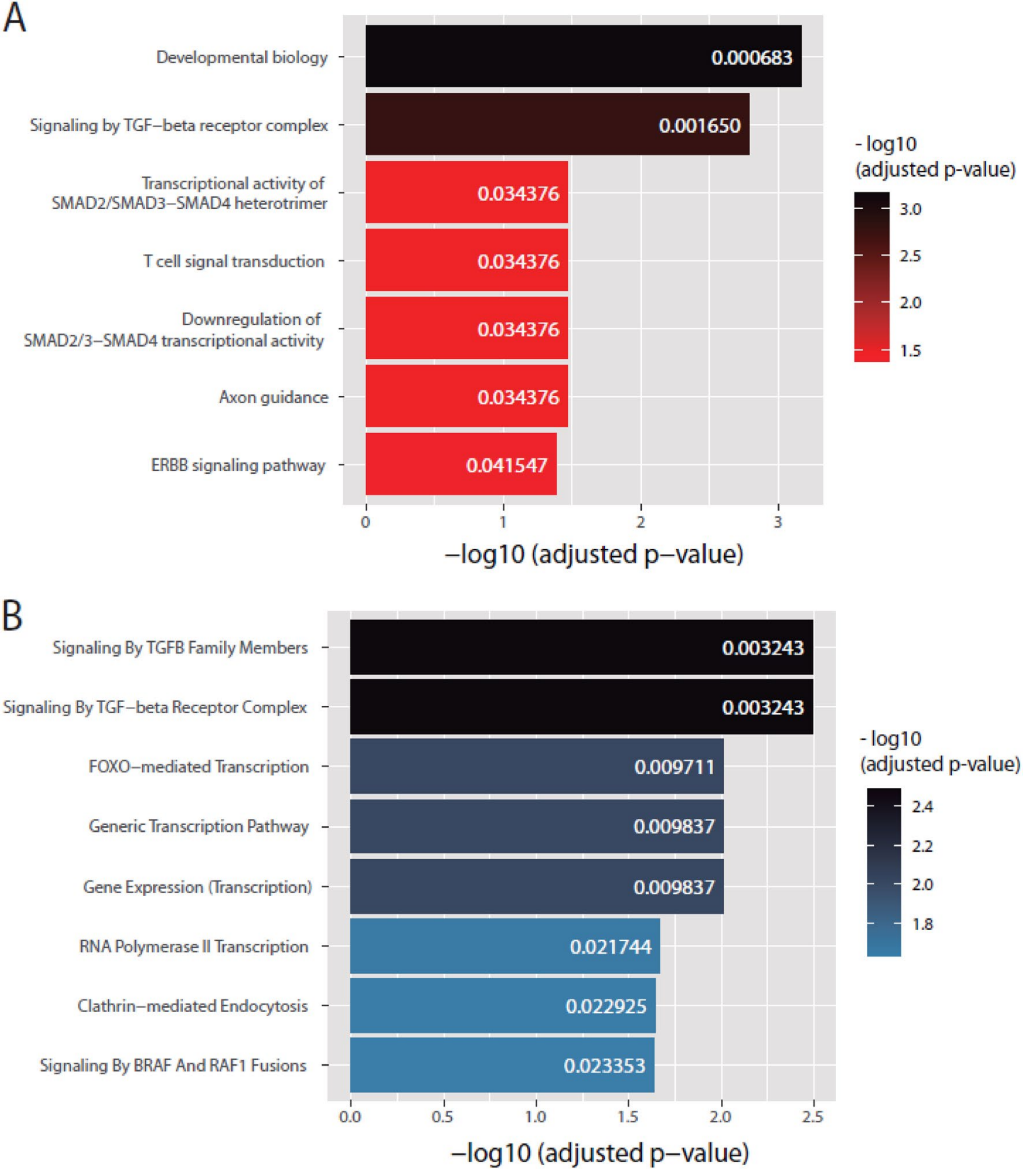
Pathways and biological processes predicted to be regulated by miR-148a-3p, miR-224-5p, miR-Contig-1519, and miR-Contig-3244. The results from the EnrichR-Bioplanet (**A**) and EnrichR-Reactome (**B**) databases are shown.

The BioPlanet database results showed that the principal targets of these four miRNAs belong to different pathways, but most of them were related to transforming growth factor (TGF)-β signaling and the transcriptional activity of the downstream heterotrimer suppressor of mothers against decapentaplegic (SMAD)2/3-SMAD4 (Fig. 5A). Additionally, some of the targets were part of ERBB signaling and T-cell signal transduction. Considering the Reactome database results (Fig. 5B), signaling of TGF-β members and receptors were the most significant biological processes. The forkhead box O (FOXO)-mediated transcription was the third most significant, and we also detected a remarkable regulation of transcriptional pathways such as those related to RNA polymerase II. Finally, these four miRNAs also regulated clathrin-mediated endocytosis, one of the ways for the parasite to enter host cells, and signaling by the serine/threonine-protein kinase B-Raf (BRAF) and the rapidly accelerated fibrosarcoma (RAF)1.

## Discussion

Previous research about the role of specific miRNAs in *T. cruzi* infection have mainly used RT-qPCR analysis of blood or cardiac tissue. In our study we have performed for the first time an exploratory small RNA-Seq analysis in the plasma of chagasic patients with different degrees of cardiac pathology, and we identified and validated by RT‒qPCR new circulating miRNAs and a miRNA signature as a potential biomarker of chronic Chagas disease. This signature is characterized by low levels of miR-Contig-1519, miR-Contig-3244, and miR-148a-3p and high levels of miR-224-5p.

Of the four novel circulating miRNAs identified, we were able to detect miR-Contig-1519 and miR-Contig-3244, and both levels significantly decreased in all the chagasic cohorts analyzed. In the case of miR-Contig-2269 and miR-Contig-5468, either technical problems involving primer design hampered their detection or because they may be short mRNAs lacking miRNA activity. Further studies are needed to probe if they are real miRNAs and/or see their effect during the *T. cruzi* infection. Considering miR-Contig-1519 and miR-Contig-3244, we confirmed their use to discriminate healthy individuals from chronic chagasic subjects, independently if they are indeterminate or patients with cardiomyopathy. Therefore, this result suggests a role of these two new miRNAs in humans that is not related to the progression of the disease but to the pathological response associated with *T. cruzi*, that should be further elucidated in posterior research considering their targets and the affected biological pathways to provide mechanistic insights into their functions.

To complete our results and establish a more powerful signature of chronic chagasic patients, we also analyzed three known miRNAs that had been described for cardiomyopathies of other etiology but not for Chagas disease: miR-148a-3p, miR-181a-5p, and miR-224-5p.

miR-148a-3p is implicated in immunopathologic conditions and could be a potential biomarker for different cardiac pathologies. According to some authors miR-148a-3p is a novel repressor of *IKBKB*, which encodes the inhibitor of nuclear factor kappa B (IκB) kinase β, that represses the nuclear factor kappa-light-chain-enhancer of activated B cells (NF-κB) signalling pathway that plays a key role in the inflammatory cascade^26^. Also, overexpression of miR-148a-3p inhibited inflammatory factors and functional injury in vascular endothelial cells^27^. Therefore, according to our data, a decrease in miR-148a-3p levels in chagasic patients could be related to an increase in endothelial and cardiac inflammation.

Moreover, miR-148a-3p affects macrophage polarization and cytokine production: it regulates the activation and polarization of mouse macrophages targeting the phosphatase and tensin homolog (PTEN) through the phosphatidylinositol 3-kinase (PI3K) and protein kinase B (AKT) pathway in response to *Schistosoma japonicum* infection^28^. Indeed, macrophage polarization is a key in the immune response against of *T. cruzi* infection^29^. Also, this miRNA is a novel downstream molecule of Notch signalling, promoting the differentiation of monocytes into macrophages by the presence of granulocyte macrophage colony-stimulating factor (GM-CSF). Specifically, it enhances M1 macrophage (activated by the classic pathway) differentiation, inhibits M2 macrophage polarization (activated by the alternative pathway) and increases the ability to engulf and kill bacteria, mediated by excessive production of reactive oxygen species (ROS) through the PTEN/AKT pathway^30^. Therefore, the reduction in miR-148a-3p in some chronic chagasic patients could inhibit M1 macrophage polarization and ROS production, both related to the elimination of the parasite and its persistence in immune cells.

We did not detect significant differences in the levels of miR-181a-5p between any of the groups, even though this miRNA is involved in cardiomyocyte apoptosis, a general problem of several cardiomyopathies^31^.

We found increased levels of miR-224-5p in ICD and MCC patients compared to the HC group. According to previous studies, this miRNA is a potential target molecule for treating and diagnosing atherosclerosis^32^. Additionally, it is increased in heart failure^33^, coronary artery disease^34^, and circulating extracellular vesicles of patients with reduced coronary flow reserve, which is related to endothelial dysfunction^35^. The upregulation of this miRNA in chronic chagasic patients, even in the absence of cardiac symptoms, is a new revelation that should be studied in the future to understand its specific role in the progression of Chagas disease.

Enrichment pathway analysis considering miR-Contig-3244, miR-Contig-1519, miR-148a-3p, and miR-224-5p targets was performed through the EnrichR webtool using the BioPlanet and Reactome databases. This analysis predicted that most of the targets are related to TGF-β signaling, including the transcriptional activity of the heterotrimer SMAD2/3-SMAD4. After their phosphorylation by TGF-β, these proteins form a heterotrimer that translocates to the nucleus to mediate intracellular TGF-β signal transduction^36^. In Chagas disease, TGF-β is involved the progression of the CC onset, promoting the heart cell invasion and intracellular replication by *T. cruzi*, host immune and the inflammation response, and posterior cardiac fibrosis. In fact, inhibitors of TGF-β signaling revert these effects and improve cardiac electric parameters in infected mice, although more studies in infected human cells or humanized animal models are needed to clarify its potential as therapeutic approach. Additionally, Chagas disease patients with higher TGF-β in serum display a worse clinical outcome suggesting a predictive value as a surrogate biomarker^37–39^. Our results support these ideas, and further studies are necessary to understand the specific role and regulation that these four miRNAs may exert on TGF-β signaling and their implication as possible biomarkers and/or therapeutic approaches for Chagas disease.

Another biological process regulated by these miRNAs was T-cell signal transduction. T cells are key for the immune response against the parasite. CD8+ T cells take part in the elimination of the parasite, although some evidence suggests that they are also involved in some of the clinical manifestations of Chagas disease, such as tissue damage and inflammatory processes. The balance between CD4+ Th1 and Th2 cells is also important during infection. Th1 cytokines contribute to the control and elimination of the parasite but produce an enhanced immune response, while Th2 cytokines are implicated in protective responses, although they allow host and parasite cell survival. However, some of the authors suggest that more research about the specific parasite targets of T cell memory response may contribute to the conceptualization and development of prophylactic or therapeutic vaccines^40–43^. Therefore, studies focusing on these specific types of cells could help to discover the role of these four miRNAs in the dilemma of T-cell implication in Chagas disease.

Additionally, our miRNAs regulate targets linked to clathrin-mediated endocytosis and RAF1/BRAF signaling. Clathrin-mediated endocytosis is one of the main ways of entry of *T. cruzi* into the host cell from the initial stages of infection until the formation of the parasitophorous vacuole, and its inhibition drastically reduces the internalization of trypomastigotes in phagocytic and epithelial cells^44^. Therefore, a critical change in this pathway may alter all the endocytic processes of the parasite and its posterior intracellular replication, although more studies with different *T. cruzi* strains and other cellular types that are infected by the parasite could help to confirm the relevance of the clathrin-mediated endocytosis in the infection process.

Regarding RAF1/BRAF signaling, both proteins are upstream kinases and activators of the mitogen-activated protein kinase (MAPK) cascade, which contains the mitogen-activated protein kinase kinase (MEK) and their targets, the extracellular signal-regulated kinase (ERK) family. Although there are not specific studies focused on RAF1 or BRAF proteins in *T. cruzi* infections, some. Some researchers studied the MAPK cascade activation and role in Chagas disease MEK/ERK pathway increase in nervous system models by the interaction of the host cells with surface molecules of *T. cruzi* as members of the multigene family of trans-sialidases^45,46^, while the inhibition of ERK1/2 phosphorylation produced a significant decrease in invasion and infection by this parasite in epithelial and cardiac muscle cells^47^ and in cultured human umbilical vein endothelial and vascular smooth muscle cells^48^. Therefore, the regulation of targets of this pathway by these miRNAs may drastically alter the infection process of *T. cruzi* and should be studied in the future to discover its real potential as a treatment for Chagas disease.

Interestingly, our results complement previous observations on miRNAs in different contexts of *T. cruzi* infection by other authors. For instance, studies in patients pointed to miR-146a as a biomarker of the indeterminate phase of Chagas disease^49^, and miRNA-155 deficiency exacerbated *T. cruzi* infection in mice, suggesting that miR-155 is an important immune regulatory molecule critical for the control of *T. cruzi* in the acute phase of the infection^50^. In addition, it has been reported that *T. cruzi* induces a differential miRNA profile in human placental explants that may be potential targets for the therapeutic control of congenital Chagas^51^. On the other hand, it has been reported that modulation of miR-145-5p and miR-146b-5p levels is linked to reduced parasite load in H9C2 *T. cruzi*-infected cardiomyoblasts, which points to a possible direct action of miRNAs in heart tissue^52^. Thus, all these reports highlight the importance of our results and previous findings by other groups about the role of miRNAs in Chagas disease.

In summary, we have identified a signature of circulating known and new miRNAs as a potential biomarker and/or therapeutic target of the chronic phase of Chagas disease. Moreover, it is important to remember that miRNA expression could vary between individuals according to both transcriptional changes in gene expression and promoter hypermethylation, and post-transcriptional changes in miRNA processing mechanisms of regulation, as well as effects of exogenous (xenobiotics) or endogenous (hormones, cytokines) compounds^53^. We recognize the limitations of our study and results (sample size, mainly), which should be considered exploratory, and therefore, these miRNAs should be further explored in a larger cohort of individuals to validate their promising use as biomarkers, therapeutic targets, or clinical treatments. In addition, more studies including severe Chagas cardiomyopathy (SCC) patients could be useful to evaluate their prognostic value. We consider it necessary for Chagas disease since it is not possible to predict whether ICD and MCC patients will develop SCC.

## Methods

### Ethics statement

This study was approved by the Bioethics Committee from the Faculty of Medical Sciences, National University of Rosario, Argentina (Resolution N° 5093/2018); the Ethical Committees of Ramón y Cajal University Hospital and *Fundación Jimenez Diaz* in Madrid (Spain, References FUN-BEN-2007-01 and 10/2016, respectively); and the Ethical Committee of Vall d’Hebrón University Hospital in Barcelona (Spain, Reference D-RTF080). All patients signed informed consent forms. Data on human subjects were analyzed anonymously, and clinical investigations were conducted according to the Declaration of Helsinki.

### Blood samples from patients

This work corresponds to an observational, cross-sectional study, where the patients and healthy volunteers were recruited from 2011 to 2017 at the Chagas disease ambulatory section from the *Hospital Provincial del Centenario* (HPC, Rosario, Argentina), in 2018 at the *Fundación Jiménez Díaz* (FJD, Madrid, Spain), and in 2020 at the Vall d’Hebrón University Hospital (HVH, Barcelona, Spain). Twenty-eight chagasic patients from Spanish hospitals were from Bolivia, 1 from Brazil, and 1 from Paraguay. Exclusion criteria comprised patients with previous treatment with Benznidazole or Nifurtimox. Healthy control subjects were seronegative for the *T. cruzi*-specific test. The diagnosis was based on at least two positive serological tests (either by ELISA, hemagglutination, or immunofluorescence), together with clinical symptoms, chest X-ray, 12-lead resting electrocardiogram (ECG), and/or echocardiography. Routine laboratory studies were also included.

A total of 66 subjects were included in the study: healthy controls (HC, n = 26) from endemic areas, indeterminate Chagas disease (ICD, n = 19), and mild and moderate Chagas cardiomyopathy (MCC, n = 21). Table S1 compiles all subjects included in the study classified by the hospital in which they were recruited. The criteria for the aggrupation of chagasic patients from HPC were as follows: ICD, symptomless, normal ECG and chest X-ray; MCC, presented no congestive heart failure with ECG showing incomplete or complete right bundle branch block, ventricular arrhythmia or normal heart size or only mild cardiomegaly (chest X-ray cardiothoracic ratio < 0.55). For patients from FJD: ICD, without pathologic alterations; and MCC, with pathologic alterations in ECG and/or echocardiogram and/or complementary tests compatible with CC. For patients from HVH: ICD, Kuschnir classification 0 and MCC, Kuschnir classification I. Individual information of the patients is shown in Supporting Data S1.

### Circulating miRNA extraction

Blood samples from patients and healthy volunteers were collected. Plasma from blood samples of HPC and HVH was obtained from EDTA-treated blood. Serum was obtained from blood samples from FJD patients. All the samples were immediately stored at − 80 °C until miRNA extraction. To avoid impacts in the circulating miRNAs profile, as other studies have demonstrated^54,55^, hemolysis processes were checked visually according to the hemolysis reference palette^56^, and all the samples used in the study were free of hemolysis. In samples from HPC and FJD, miRNAs were extracted using a QIAamp miRNeasy Serum/plasma kit (Qiagen, Germantown, USA), and samples from HVH were extracted using a miRNeasy Serum/Plasma Advance Kit (Qiagen). A spike-in mix (Qiagen, RNA Spike-In Kit containing sp2, sp4, and sp5) was added as a control for sample quality and PCR efficiency. The procedures were carried out following the directions of the manufacturer. Small RNA-Seq was performed using samples from HPC, and two or three extractions of each sample were performed to obtain at least 25 ng per sample. Then, the different extractions of each sample were gathered together. The amount of RNA was quantified using RiboGreen. Pools were made for each group (HC, ICD, and MCC), and each group was composed of 5 samples. The pools were concentrated to obtain at least 25 ng in a final volume of 6 µl, which passed the quality assessment in the bioanalyzer and were sent for sequencing to the *Parque Científico de Madrid* (Madrid, Spain).

### Small RNA-Seq analysis

The total RNA fraction corresponding to miRNAs (15-30 bases) was purified by size exclusion in polyacrylamide gels and the miRNeasy Micro Kit (Qiagen Cat. No. 217084). Subsequently, the sequencing libraries were prepared with the TruSeq kit (Illumina) for Small RNA-Seq analysis following the specific protocols of the Illumina technology (TruSeq) without modifications by the Genomics facility of the *Parque Científico de Madrid* (Madrid, Spain), and readings of 14-50 bases in length were generated in a single replicate using MiSeq technology (Illumina). Differential expression analysis and miRNA *de novo* finding were performed using the DEseq2^57^ and miRDeep2^58^ packages, respectively.

### miRNA analysis by RT‒qPCR

RT‒qPCR analysis was performed at the *Parque Científico de Madrid* (Madrid, Spain). cDNA synthesis was performed with the miRCURY LNA RT Kit (Qiagen Cat. No. 339340) according to the manufacturer’s instructions. Then, 2 µl of purified total miRNAs (10 ng) from plasma or serum was used as input RNA and mixed with 0.5 µL synthetic RNA spike-in (sp6) before reverse transcription. The obtained cDNAs were diluted 1/40 in RNAse-free water before qPCR amplification. Real-time PCR amplification was performed using the miRCURY SYBR Green PCR Kit (Qiagen Cat. No. 339347) in the presence of PCR Locked Nucleic Acid (LNA) primers specific for spike-in sp2, sp4, sp5, and sp6, miR-103a-3p, miR-Contig-5468, miR-Contig-1519, miR-Contig-2269, miR-Contig-3244, miR-148a-3p, miR-224-5p and miR-181a-5p (Qiagen). Three replicates were run for each combination of genes and samples. To obtain Cp values, we used LightCycler® 480 Software following the 2nd derivative max method. The expression levels of target miRNAs were normalized to the expression of endogenous miR-103a-3p. The results refer to HC samples. Relative changes and statistical values of miRNA expression levels were calculated by the ΔΔC_t_ method using LightCycler® 480 Software without correction for assay efficiency. The results were expressed as log_10_RQ (RQ = 2^–ΔΔCt^).

### Statistical analysis of the small RNA-Seq and the RT‒qPCR

The main limitation of the present study is the lack of technical replicates in small RNA-Seq; therefore, Basemean (normalized reads) was chosen as an additional filter to the adjusted p value as a measure of miRNA abundance. miRNAs with less than 100 normalized reads in the healthy controls and the patient samples were discarded for further analysis. All PCAs were performed using the R programming language (version 4.2.2). Bar plots and statistical significance were evaluated using GraphPad Prism (Version 8.0). After the Shapiro‒Wilk normality test, two-way ANOVA tests were applied as indicated in the figure legends (*p<0.05, **p<0.01, ***p<0.001, ****p<0.0001).

### Pathway analysis of the relevant miRNA targets

Target prediction of the most significant miRNAs in our study (miR-148a-3p, miR-224-5p, and miR-Contig-1519) was performed with the miRDB web tool^59,60^. Targets with a score equal to or higher than 90 were used for enrichment pathway analysis in the EnrichR web tool using the BioPlanet and Reactome databases^22,23^.

### Supplementary Information

Supplementary Information 1.

Supplementary Information 2.

Supplementary Information 3.

Supplementary Information 4.

Supplementary Information 5.

## Data Availability

All raw sequencing data generated from human plasma in this study are accessible in the National Center for Biotechnology Information (https://www.ncbi.nlm.nih.gov/) under the BioProject PRJNA853048, with the following BioSample accessions: SAMN29358576, SAMN29358577, SAMN29358578, and SAMN29358579. Temporary submission ID: SUB11686132; release date: 2024-03-30, or after publication, whichever is first.

## References

[1] Pérez-Molina, J. A. & Molina, I. Chagas disease. *Lancet* **391**, 82–94 (2018). 28673423 10.1016/S0140-6736(17)31612-4

[2] Prata, A. Clinical and epidemiological aspects of Chagas disease. *Lancet Infect. Dis.* **1**, 92–100 (2001). 11871482 10.1016/S1473-3099(01)00065-2

[3] Rassi, A., Rassi, A. & Marin-Neto, J. A. Chagas disease. *Lancet* **375**, 1388–1402 (2010). 20399979 10.1016/S0140-6736(10)60061-X

[4] Blondal, T. *et al.* Assessing sample and miRNA profile quality in serum and plasma or other biofluids. *Methods* **59**, S1-6 (2013). 23036329 10.1016/j.ymeth.2012.09.015

[5] Laugier, L. *et al.* miRNAs may play a major role in the control of gene expression in key pathobiological processes in Chagas disease cardiomyopathy. *PLoS Negl. Trop. Dis.* **14**, e0008889 (2020). 33351798 10.1371/journal.pntd.0008889 PMC7787679

[6] Murchison, E. P. & Hannon, G. J. miRNAs on the move: miRNA biogenesis and the RNAi machinery. *Curr. Opin. Cell Biol.* **16**, 223–229 (2004). 15145345 10.1016/j.ceb.2004.04.003

[7] Bronze-da-Rocha, E. MicroRNAs expression profiles in cardiovascular diseases. *Biomed. Res. Int.* **2014**, 985408 (2014). 25013816 10.1155/2014/985408 PMC4075084

[8] de Gonzalo-Calvo, D., Iglesias-Gutiérrez, E. & Llorente-Cortés, V. Epigenetic biomarkers and cardiovascular disease: Circulating MicroRNAs. *Rev. Esp. Cardiol. (Engl. Ed.)* **70**, 763–769 (2017). 28623159 10.1016/j.rec.2017.05.013

[9] Sayed, D., Hong, C., Chen, I.-Y., Lypowy, J. & Abdellatif, M. MicroRNAs play an essential role in the development of cardiac hypertrophy. *Circ. Res.* **100**, 416–424 (2007). 17234972 10.1161/01.RES.0000257913.42552.23

[10] van Empel, V. P. M., De Windt, L. J. & da Costa Martins, P. A. Circulating miRNAs: Reflecting or affecting cardiovascular disease?. *Curr. Hypertens. Rep.* **14**, 498–509 (2012). 22996205 10.1007/s11906-012-0310-7

[11] van Rooij, E. *et al.* Dysregulation of microRNAs after myocardial infarction reveals a role of miR-29 in cardiac fibrosis. *Proc. Natl. Acad. Sci. U.S.A.* **105**, 13027–13032 (2008). 18723672 10.1073/pnas.0805038105 PMC2529064

[12] Correia, C. N. *et al.* Circulating microRNAs as potential biomarkers of infectious disease. *Front. Immunol.* **8**, 118 (2017). 28261201 10.3389/fimmu.2017.00118 PMC5311051

[13] Miao, C., Chang, J., Zhang, G. & Fang, Y. MicroRNAs in type 1 diabetes: New research progress and potential directions. *Biochem. Cell Biol.* **96**, 498–506 (2018). 29554441 10.1139/bcb-2018-0027

[14] Lopez-Pedrera, C. *et al.* Role of microRNAs in the development of cardiovascular disease in systemic autoimmune disorders. *Int. J. Mol. Sci.* **21**, E2012 (2020). 10.3390/ijms21062012 PMC7139533 32188016

[15] Jones Buie, J. N., Goodwin, A. J., Cook, J. A., Halushka, P. V. & Fan, H. The role of miRNAs in cardiovascular disease risk factors. *Atherosclerosis* **254**, 271–281 (2016). 27693002 10.1016/j.atherosclerosis.2016.09.067 PMC5125538

[16] Moldovan, L. *et al.* Methodological challenges in utilizing miRNAs as circulating biomarkers. *J. Cell. Mol. Med.* **18**, 371–390 (2014). 24533657 10.1111/jcmm.12236 PMC3943687

[17] Linhares-Lacerda, L. *et al.* Circulating plasma MicroRNA-208a as potential biomarker of chronic indeterminate phase of Chagas disease. *Front. Microbiol.* **9**, 269 (2018). 29559958 10.3389/fmicb.2018.00269 PMC5845676

[18] Nonaka, C. K. V. *et al.* Circulating miRNAs as potential biomarkers associated with cardiac remodeling and fibrosis in chagas disease cardiomyopathy. *Int. J. Mol. Sci.* **20**, E4064 (2019). 10.3390/ijms20164064 PMC6721092 31434314

[19] Ferreira, L. R. P. *et al.* MicroRNAs miR-1, miR-133a, miR-133b, miR-208a and miR-208b are dysregulated in chronic Chagas disease cardiomyopathy. *Int. J. Cardiol.* **175**, 409–417 (2014). 24910366 10.1016/j.ijcard.2014.05.019

[20] Friedländer, M. R. *et al.* Discovering microRNAs from deep sequencing data using miRDeep. *Nat. Biotechnol.* **26**, 407–415 (2008). 18392026 10.1038/nbt1394

[21] O’Brien, J., Hayder, H., Zayed, Y. & Peng, C. Overview of MicroRNA biogenesis, mechanisms of actions, and circulation. *Front. Endocrinol.*10.3389/fendo.2018.00402 (2018). 10.3389/fendo.2018.00402 PMC6085463 30123182

[22] Kuleshov, M. V. *et al.* Enrichr: A comprehensive gene set enrichment analysis web server 2016 update. *Nucleic Acids Res.* **44**, W90-97 (2016). 27141961 10.1093/nar/gkw377 PMC4987924

[23] Xie, Z. *et al.* Gene set knowledge discovery with Enrichr. *Curr. Protoc.* **1**, e90 (2021). 33780170 10.1002/cpz1.90 PMC8152575

[24] Huang, R. *et al.* The NCATS BioPlanet—An integrated platform for exploring the universe of cellular signaling pathways for toxicology, systems biology, and chemical genomics. *Front. Pharmacol.* **10**, 445 (2019). 31133849 10.3389/fphar.2019.00445 PMC6524730

[25] Gao, W. *et al.* Multiplex indexing approach for the detection of DNase I hypersensitive sites in single cells. *Nucleic Acids Res.* **49**, e56 (2021). 33693880 10.1093/nar/gkab102 PMC8191781

[26] Patel, V. *et al.* The stretch responsive microRNA miR-148a-3p is a novel repressor of IKBKB, NF-κB signaling, and inflammatory gene expression in human aortic valve cells. *FASEB J.* **29**, 1859–1868 (2015). 25630970 10.1096/fj.14-257808 PMC4771066

[27] Wang, F. *et al.* KLF5/LINC00346/miR-148a-3p axis regulates inflammation and endothelial cell injury in atherosclerosis. *Int. J. Mol. Med.* **48**, 1–10 (2021). 10.3892/ijmm.2021.4985 34165154

[28] Giri, B. R. & Cheng, G. Host miR-148 regulates a macrophage-mediated immune response during *Schistosoma**japonicum* infection. *Int. J. Parasitol.* **49**, 993–997 (2019). 31726056 10.1016/j.ijpara.2019.08.002

[29] Aoki, M. P. *et al.* Cruzipain, a major *Trypanosoma**cruzi* antigen, promotes arginase-2 expression and survival of neonatal mouse cardiomyocytes. *Am. J. Physiol. Cell Physiol.* **286**, C206-212 (2004). 13679306 10.1152/ajpcell.00282.2003

[30] Huang, F. *et al.* miR-148a-3p mediates notch signaling to promote the differentiation and M1 activation of macrophages. *Front. Immunol.* **8**, 1327 (2017). 29085372 10.3389/fimmu.2017.01327 PMC5650608

[31] Cheng, Y. *et al.* Inhibition of long non-coding RNA metastasis-associated lung adenocarcinoma transcript 1 attenuates high glucose-induced cardiomyocyte apoptosis via regulation of miR-181a-5p. *Exp. Anim.* **69**, 34–44 (2020). 31353329 10.1538/expanim.19-0058 PMC7004813

[32] Zhai, C. *et al.* LncRNA AK087124/miR-224-5p/PTEN axis modulates endothelial cell injury in atherosclerosis through apoptosis and AKT signaling pathway. *Arch. Biochem. Biophys.* **705**, 108916 (2021). 33974917 10.1016/j.abb.2021.108916

[33] Zheng, H., Shi, L., Tong, C., Liu, Y. & Hou, M. circSnx12 is involved in ferroptosis during heart failure by targeting miR-224-5p. *Front. Cardiovasc. Med.* **8**, 656093 (2021). 33969020 10.3389/fcvm.2021.656093 PMC8097164

[34] Levstek, T., Karun, T., Rehberger Likozar, A., Šebeštjen, M. & Trebušak Podkrajšek, K. Interplay between microRNAs, serum proprotein convertase subtilisin/kexin type 9 (PCSK9), and lipid parameters in patients with very high lipoprotein(a) treated with PCSK9 inhibitors. *Genes (Basel)* **14**, 632 (2023). 36980904 10.3390/genes14030632 PMC10048228

[35] James, K. *et al.* Increased expression of miR-224-5p in circulating extracellular vesicles of patients with reduced coronary flow reserve. *BMC Cardiovasc. Disord.* **22**, 321 (2022). 35850658 10.1186/s12872-022-02756-w PMC9290204

[36] Nakao, A. *et al.* TGF-beta receptor-mediated signalling through Smad2, Smad3 and Smad4. *EMBO J.* **16**, 5353–5362 (1997). 9311995 10.1093/emboj/16.17.5353 PMC1170167

[37] Ferreira, R. R. *et al.* The search for biomarkers and treatments in Chagas disease: Insights from TGF-beta studies and immunogenetics. *Front. Cell. Infect. Microbiol.* **11**, 767576 (2021). 35186778 10.3389/fcimb.2021.767576 PMC8847772

[38] Waghabi, M. C. *et al.* Transforming growth factor-ß as a therapeutic target for the cardiac damage of Chagas disease. *Mem. Inst. Oswaldo Cruz* **117**, e210395 (2022). 35239842 10.1590/0074-02760210395 PMC8896758

[39] Ferreira, R. R. *et al.* In Chagas disease, transforming growth factor beta neutralization reduces *Trypanosoma**cruzi* infection and improves cardiac performance. *Front. Cell. Infect. Microbiol.* **12**, 1017040 (2022). 36530434 10.3389/fcimb.2022.1017040 PMC9748701

[40] Ferragut, F., Acevedo, G. R. & Gómez, K. A. T cell specificity: A great challenge in Chagas disease. *Front. Immunol.* **12**, 674078 (2021). 34267750 10.3389/fimmu.2021.674078 PMC8276045

[41] Acevedo, G. R., Girard, M. C. & Gómez, K. A. The unsolved Jigsaw puzzle of the immune response in Chagas disease. *Front. Immunol.* **9**, 1929 (2018). 30197647 10.3389/fimmu.2018.01929 PMC6117404

[42] Cuervo, H. *et al.* Inducible nitric oxide synthase and arginase expression in heart tissue during acute *Trypanosoma**cruzi* infection in mice: Arginase I is expressed in infiltrating CD68+ macrophages. *J. Infect. Dis.* **197**, 1772–1782 (2008). 18473687 10.1086/529527

[43] Poveda, C. *et al.* Interaction of signaling lymphocytic activation molecule family 1 (SLAMF1) receptor with *Trypanosoma**cruzi* is strain-dependent and affects NADPH oxidase expression and activity. *PLoS Negl. Trop. Dis.* **14**, e0008608 (2020). 32925918 10.1371/journal.pntd.0008608 PMC7515593

[44] Barrias, E., Reignault, L., de Carvalho, T. M. U. & de Souza, W. Clathrin coated pit dependent pathway for *Trypanosoma**cruzi* internalization into host cells. *Acta Trop.* **199**, 105057 (2019). 31202818 10.1016/j.actatropica.2019.105057

[45] Chuenkova, M. V. & Pereira, M. A. The *T*. *Cruzi* trans-sialidase induces PC12 cell differentiation via MAPK/ERK pathway. *Neuroreport* **12**, 3715–3718 (2001). 11726780 10.1097/00001756-200112040-00022

[46] Lattanzi, R., Maftei, D., Fullone, M. R. & Miele, R. *Trypanosoma**cruzi* trans-sialidase induces STAT3 and ERK activation by prokineticin receptor 2 binding. *Cell Biochem. Funct.* **39**, 326–334 (2021). 32892338 10.1002/cbf.3586

[47] Ferri, G., Musikant, D. & Edreira, M. M. Host cell Rap1b mediates cAMP-dependent invasion by *Trypanosoma**cruzi*. *PLoS Negl. Trop. Dis.* **17**, e0011191 (2023). 36897926 10.1371/journal.pntd.0011191 PMC10032529

[48] Mukherjee, S. *et al.**Trypanosoma**cruzi* infection activates extracellular signal-regulated kinase in cultured endothelial and smooth muscle cells. *Infect. Immun.* **72**, 5274–5282 (2004). 15322023 10.1128/IAI.72.9.5274-5282.2004 PMC517449

[49] Ballinas-Verdugo, M. A. *et al.* Circulating miR-146a as a possible candidate biomarker in the indeterminate phase of Chagas disease. *Biol. Res.* **54**, 21 (2021). 34289913 10.1186/s40659-021-00345-3 PMC8293491

[50] Jha, B. K. *et al.* MicroRNA-155 deficiency exacerbates *Trypanosoma**cruzi* infection. *Infect. Immun.*10.1128/IAI.00948-19 (2020). 32312766 10.1128/IAI.00948-19 PMC7309613

[51] Medina, L. *et al.**Trypanosoma**cruzi* and *Toxoplasma**gondii* induce a differential MicroRNA profile in human placental explants. *Front. Immunol.* **11**, 595250 (2020). 33240284 10.3389/fimmu.2020.595250 PMC7677230

[52] Farani, P. S. G., Ferreira, B. I. S., Gibaldi, D., Lannes-Vieira, J. & Moreira, O. C. Modulation of miR-145-5p and miR-146b-5p levels is linked to reduced parasite load in H9C2 *Trypanosoma**cruzi* infected cardiomyoblasts. *Sci. Rep.* **12**, 1436 (2022). 35082354 10.1038/s41598-022-05493-4 PMC8791985

[53] Gulyaeva, L. F. & Kushlinskiy, N. E. Regulatory mechanisms of microRNA expression. *J. Transl. Med.* **14**, 143 (2016). 27197967 10.1186/s12967-016-0893-x PMC4873990

[54] Pizzamiglio, S. *et al.* A methodological procedure for evaluating the impact of hemolysis on circulating microRNAs. *Oncol. Lett.* **13**, 315–320 (2017). 28123561 10.3892/ol.2016.5452 PMC5244842

[55] Kirschner, M. B. *et al.* The impact of hemolysis on cell-free microRNA biomarkers. *Front. Genet.* **4**, 94 (2013). 23745127 10.3389/fgene.2013.00094 PMC3663194

[56] A Quick-Reference Tool for Hemolysis Status | Division of Vector-Borne Diseases | NCEZID | CDC. https://www.cdc.gov/ncezid/dvbd/specimensub/hemolysis-palette.html (2023).

[57] Love, M. I., Huber, W. & Anders, S. Moderated estimation of fold change and dispersion for RNA-seq data with DESeq2. *Genome Biol.* **15**, 550 (2014). 25516281 10.1186/s13059-014-0550-8 PMC4302049

[58] Friedländer, M. R., Mackowiak, S. D., Li, N., Chen, W. & Rajewsky, N. miRDeep2 accurately identifies known and hundreds of novel microRNA genes in seven animal clades. *Nucleic Acids Res.* **40**, 37–52 (2012). 21911355 10.1093/nar/gkr688 PMC3245920

[59] Chen, Y. & Wang, X. miRDB: An online database for prediction of functional microRNA targets. *Nucleic Acids Res.* **48**, D127–D131 (2020). 31504780 10.1093/nar/gkz757 PMC6943051

[60] Liu, W. & Wang, X. Prediction of functional microRNA targets by integrative modeling of microRNA binding and target expression data. *Genome Biol.* **20**, 18 (2019). 30670076 10.1186/s13059-019-1629-z PMC6341724

