# Author Correction: Discovery of circulating miRNAs as biomarkers of chronic Chagas heart disease via a small RNA-Seq approach

**DOI:** 10.1038/s41598-024-69310-w

**Published:** 2024-08-09

**Authors:** Silvina R. Villar, Alfonso Herreros-Cabello, Francisco Callejas-Hernández, María C. Maza, Javier del Moral-Salmoral, Mario Gómez-Montes, Héctor O. Rodríguez-Angulo, Irene Carrillo, Miguel Górgolas, Pau Bosch-Nicolau, Israel Molina, José A. Pérez-Molina, Begoña Monge-Maillo, Oscar A. Bottasso, Juan Beloscar, Ana R. Pérez, Manuel Fresno, Núria Gironès

**Affiliations:** 1 Instituto de Inmunología Clínica y Experimental de Rosario (IDICER-CONICET-UNR), Rosario, Argentina; 2 Departamento de Biología Molecular, Universidad Autónoma de Madrid (UAM), 28049 Madrid, Spain; 3 Centro de Biología Molecular Severo Ochoa (CSIC-UAM), Madrid, Spain; 4 Instituto Venezolano de Investigaciones Científicas- IVIC, Caracas, 1021 Venezuela; 5 Division of Infectious Diseases, IIS-Fundación Jiménez Díaz, Madrid, Spain; 6 Department of Medicine, Universidad Autónoma de Madrid, Madrid, Spain; 7 International Health Unit Vall d’Hebron‑Drassanes, Infectious Diseases Department, Vall d’Hebron University Hospital, PROSICS Barcelona, Barcelona, Spain; 8 Centro de Investigación Biomédica en Red de Enfermedades Infecciosas (CIBERINFEC), Instituto de Salud Carlos III, Madrid, Spain; 9 National Referral Unit for Tropical Diseases, Infectious Diseases Department, Ramón y Cajal University Hospital, IRICYS, Madrid, Spain; 10 CIBER de Enfermedades Infecciosas, Instituto de Salud Carlos III, Madrid, Spain; 11 Cátedra y Servicio de Cardiología, Sección Chagas, Hospital Provincial del Centenario, Rosario, Argentina; 12 Instituto Universitario de Biología Molecular, Universidad Autónoma de Madrid (IUBM-UAM), Madrid, Spain; 13 Instituto de Investigación Sanitaria, Hospital Universitario de La Princesa, Madrid, Spain; 14 Present Address: Center for Genomics and Systems Biology, Department of Biology, New York University, New York, NY USA

Correction to: *Scientific Reports* 10.1038/s41598-024-51487-9, published online 12 January 2024

The Funding section in the original version of this Article was incomplete.

“The funding was supported by Agencia Nacional e Promoción Científica y Tecnológica, PICT 2013-1892, Secretaría de Ciencia y Tecnología, Universidad Nacional de Rosario, 1MED410, Ministerio de Economía y competitividad and Fondo Europeo de Desarrollo Regional, SAF2016-75988-R (MINECO/FEDER), SAF2015-63868-R (MINECO/FEDER), Red de Investigación de Centros de Enfermedades Tropicales, RICET RD12/0018/0004, Consejería de Sanidad, Comunidad de Madrid, S-2010/BMD-2332, Ministerio de Ciencia, Innovación y Universidades-Agencia Estatal de Investigación and Fondo Europeo de Desarrollo Regional, PGC2018-096132-B-I00.”

now reads:

“The funding was supported by Agencia Nacional e Promoción Científica y Tecnológica, PICT 2013-1892, Secretaría de Ciencia y Tecnología, Universidad Nacional de Rosario, 1MED410, Ministerio de Economía y competitividad and Fondo Europeo de Desarrollo Regional, SAF2016-75988-R (MINECO/FEDER), SAF2015-63868-R (MINECO/FEDER), Red de Investigación de Centros de Enfermedades Tropicales, RICET RD12/0018/0004, Consejería de Sanidad, Comunidad de Madrid, S-2010/BMD-2332, Ministerio de Ciencia, Innovación y Universidades-Agencia Estatal de Investigación and Fondo Europeo de Desarrollo Regional, PGC2018-096132-B-I00, and PID2021-123389OB-I00.”

The original Article has been corrected.

