# Supplementary material for: Discovery of circulating miRNAs as biomarkers of chronic Chagas heart disease via a small RNA-Seq approach
Source: Sci Rep. 2024 Jan 12;14:1187. doi: 10.1038/s41598-024-51487-9 (PMC10786931; doi:10.1038/s41598-024-51487-9)
Supplement: Supplementary file 5 — Supplementary Information 5. [file 41598_2024_51487_MOESM5_ESM.docx]

Supplementary information

# Supporting Data

The following data will be uploaded in excel format:

S1_clinical data

S2_mds_mirbase_nomlzd_reads

S3_target_prediction_1519_3244_148a_224

S4_list_bioplanet_reactome_data

# Supplementary Figures and Tables

## Supplementary Tables

**Table S1. Patients included in the study**. Number, percent of men, and mean age of HC, ICD and MCC patients recruited at HPC, FJD and HVH. miRNAs samples from HPC patients were used for small RNA-Seq while samples from FJD and HVH patients were used for the RT-qPCR studies.

|  | **Hospital of origin** | | | | | | | | |  |
| --- | --- | --- | --- | --- | --- | --- | --- | --- | --- | --- |
|  | **HPC (Plasma)** | | | **FJD (Serum)** | | | **HVH (Plasma)** | | |  |
| **Groups of patients** | **n** | **Men (%)** | **Age (mean)** | **n** | **Men (%)** | **Age (mean)** | **n** | **Men (%)** | **Age (mean)** | **Total (n)** |
| **HC** | **5** | 20 | 42 | **9** | 36 | 31 | **12** | 50 | 37 | **26** |
| **ICD** | **5** | 60 | 47 | **11** | 18 | 37 | **3** | 33 | 38 | **19** |
| **MCC** | **5** | 20 | 52 | **10** | 20 | 53 | **6** | 50 | 50 | **21** |
| **Total** | **15** |  |  | **30** |  |  | **21** |  |  | **66** |

**Table S2. Small RNA-Seq alignment to the human genome.** The number of the total reads and percentage of reads aligned at least once to the human genome found for each pool and total (mean).

| **Pools** | **Total**  **reads** | **Mapped reads (%)** |
| --- | --- | --- |
| **HC** | 10,389,423 | 99.00 |
| **ICD** | 10,640,346 | 98.84 |
| **MCC** | 10,441,248 | 98.82 |
| **Total (mean)** | 10,252,966 | 98.95 |

**Table S3.** Summary of miRDeep2 new miRNAS detection. Clusters correspond to potentially new miRNA precursors and/or mature sequences.

| **Sample** | **miRNAs** | **Clusters** |
| --- | --- | --- |
| **HC** | 258 | 204 |
| **ICD** | 274 | 157 |
| **MCC** | 338 | 185 |
| **Total** | 1331 | 806 |

**Table S4.** Data summary of predicted new miRNAs with miRDeep2 after filtering according to ranfold p-value, star sequence, precursor, and loop. Cluster 18 corresponds to sequences mapped to chromosome 19; cluster 32 to chromosome 3; cluster 128 to chromosome 5 and cluster 304 to chromosome 7. Species abbreviations: oan, *Ornithorhynchus anatinus*; has, *Homo sapiens*; dme, *Drosophila melanogaster*; gma, *Glycine max.*

## Supplementary figures


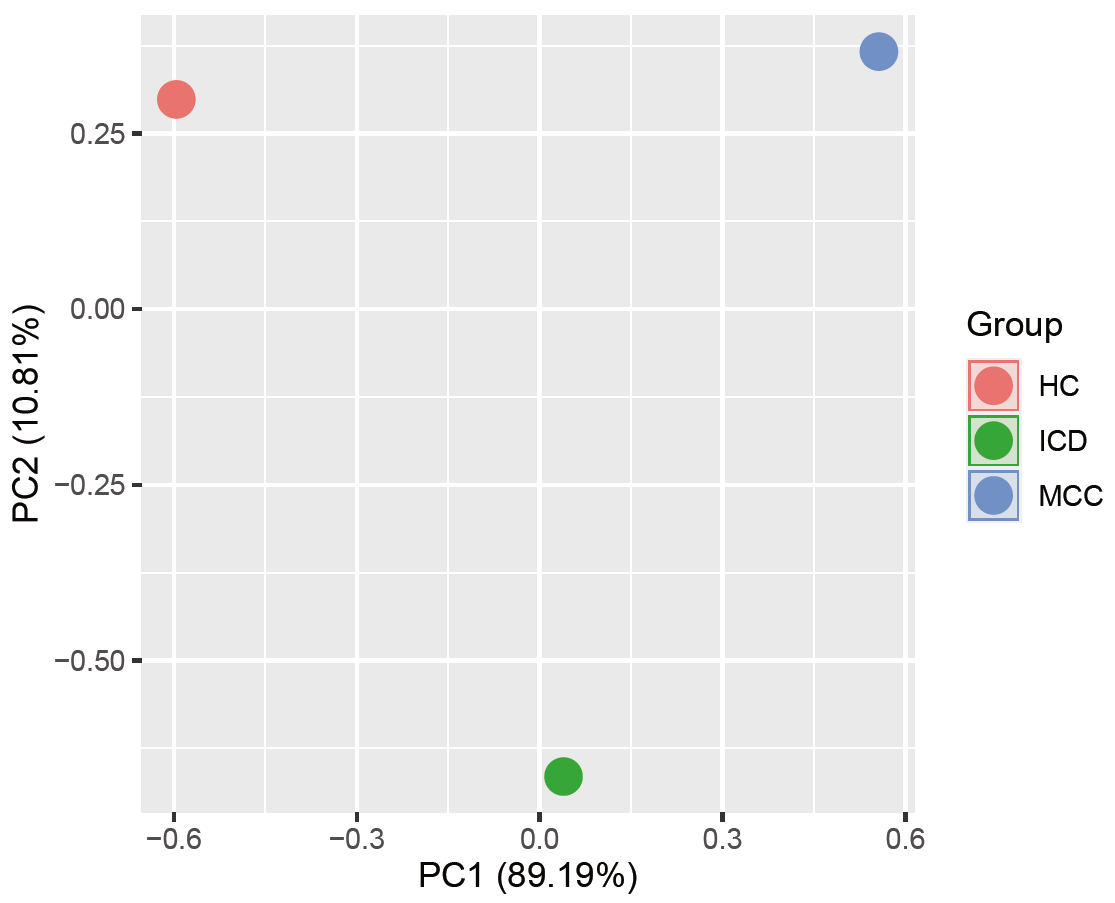


**Figure S1.** Principal Component Analysis of the normalized reads of the miRNAs identified by the Small RNA-Seq. Healthy controls (HC), Indeterminate Chagas Disease (ICD) and Mild Chagas Cardiomyopathy (MCC) groups are displayed.
